# Optimal Security Protection Strategy Selection Model Based on Q-Learning Particle Swarm Optimization

**DOI:** 10.3390/e24121727

**Published:** 2022-11-25

**Authors:** Xin Gao, Yang Zhou, Lijuan Xu, Dawei Zhao

**Affiliations:** Shandong Provincial Key Laboratory of Computer Networks, Shandong Computer Science Center (National Supercomputer Center in Jinan), Qilu University of Technology (Shandong Academy of Sciences), Jinan 250014, China

**Keywords:** Bayesian attack graph, optimal protection strategy, Q-Learning, particle swarm optimization

## Abstract

With the rapid development of Industrial Internet of Things technology, the industrial control system (ICS) faces more and more security threats, which may lead to serious risks and extensive damage. Naturally, it is particularly important to construct efficient, robust, and low-cost protection strategies for ICS. However, how to construct an objective function of optimal security protection strategy considering both the security risk and protection cost, and to find the optimal solution, are all significant challenges. In this paper, we propose an optimal security protection strategy selection model and develop an optimization framework based on Q-Learning particle swarm optimization (QLPSO). The model performs security risk assessment of ICS by introducing the protection strategy into the Bayesian attack graph. The QLPSO adopts the Q-Learning to improve the local optimum, insufficient diversity, and low precision of the PSO algorithm. Simulations are performed on a water distribution ICS, and the results verify the validity and feasibility of our proposed model and the QLPSO algorithm.

## 1. Introduction

An industrial control system refers to the equipment, system, network, and controller applied in industrial production, whose main functions are to operate, control, and assist industrial automation production [1,2,3]. ICS is mainly divided into four parts: Supervisor Control And Data Acquisition (SCADA) [4], Distributed Control System (DCS) [5], Programmable Logic Controller (PLC) [6], and Process Control System (PCS) [7]. ICS is widely used in electric power, nuclear power plants, petroleum, and other industries [8,9]. Due to the increasingly close connection between ICS and the Internet, ICS often faces complex network attacks, which pose a huge threat to the social economy and people’s security. In recent years, cyber-attack incidents against ICS have occurred frequently, such as the Iranian “Stuxnet” incident in 2010 [10] and the Ukraine power grid cyber-attack in 2015 [11], causing serious property damage. However, there are essential differences between ICS and IT, which results in the unavailability of the traditional IT security protection strategies, such as access control, firewall, and so on, for the protection of ICS [12,13,14,15].

Recently, a hot research topic in the protection strategies for ICS has been to focus on the active protection, which implements active protection strategies based on the risk assessment on the current security risk of the ICS. Shameli-Sendi et al. proposed a retrospective burst response method based on an adaptive and cost-sensitive model [16]. This method takes into account the effectiveness of the application response. However, this approach does not meet the security requirements of ICS well. K. Deb et al. proposed the non-dominated sorting GA algorithm, which can keep the diversity of solutions at a high level [17]. Genetic algorithms generate defense and attack strategies, while fitness functions are used to infer dominant strategies. This method can maintain a high accuracy, but it will fall into the local optimal and cannot solve the optimal solution. Granadillo et al. proposed a geometric model to select a best-response combination based on the response Return on Investment (RORI) index [18]. However, this approach ignores attack modeling. Attack modeling is critical because cyber attacks are becoming more destructive these days, and the response time is the determining factor. Miehling et al. used the concept of POMDP to model defense issues [19]. Their goal is to provide the best dynamic defense strategy against ongoing cyber attacks against protected systems. However, this approach uses only a single metric: the cost of deployment costs of attack or defense operations, and cannot quantify the problem well.

In the stage of implementing protection strategies, each protection strategy is accompanied with costs. How to balance the benefits and costs of protection under the constraint of limited resources is a typical optimization problem. Constructing such a reasonable optimization problem and finding its optimal solution are the main challenges.

In this paper, we propose an optimal security protection strategy selection model based on QLPSO. In order to evaluate the security situation of ICS, we introduce the protection strategy into the Bayesian attack graph, and calculate the probability of each attribute node being attacked to obtain the risk value. When choosing the optimal protection strategy, the general PSO algorithm often falls into a local optimum. To solve this problem, QLPSO is proposed, which updates the parameters of PSO algorithm through Q-Learning. Finally, we verify the validity and feasibility of our model and the QLPSO algorithm for a water distribution ICS.

## 2. Related Work

A lot of research has been done on constructing the optimal security protection strategy for various complex systems. Jaquith proposed security metrics, such as attack cost, defense implementation cost, attack impact, operation cost, and other indicators to define the factors of optimal solution [20]. However, this approach lacks specific and commonly used measurement systems to reliably evaluate countermeasures. S. Bandyopadhyay et al. proposed the single-objective optimization problem and multi-objective optimization problem to determine the optimal strategy [21]. Nevertheless, they did not discuss how to find the optimal strategy. Poolsappasit et al. proposed a multi-index quantitative analysis method based on cost and benefit, and calculated the optimal security protection strategy through genetic algorithm [22], while it is also easy to fall into the local optimum. Yigit et al. developed a network defense algorithm under limited budget conditions [23]. However, the algorithm only considers the minimum cost, does not consider the attack benefit, and lacks comprehensive measurement. Lei et al. developed a Markov game-based strategy selection mechanism for a balance between the defensive revenue and network service quality [24]. However, the method has high time cost. Herold et al. defined the response selection approach based on user-defined cost metrics to counteract security incidents in complex network environments [25]. However, this method does not meet the ICS security requirements well due to the ignorance of the balance between security risks and protection costs. S. A. Butler proposed a multi-attribute risk assessment framework, in which several complex metrics are introduced, such as total security control cost, attack strategy cost, and so on [26,27]. Roy et al. proposed a cost-effective countermeasure system for cyber attacks using various functions to form the objective function [28]. Viduto et al. proposed a new risk assessment and optimization model to solve the problem of security countermeasure selection, in which the total cost of security control and the total risk level of the system together constitute the objective function [29]. In this approach, historical databases are used without consideration for the first experienced or zero-day attacks, exposing the system to undue risk. R. Dewri proposed Genetic Algorithm (GA) and S. Wang et al. proposed Ant Colony Optimization (ACO), which utilizes single-objective and multi-objective optimization cost functions to select the optimal set of strategies [30,31]. Similarly, the indicators used in the methodology are inconsistent with globally accepted standards. B. Kordy et al. proposed a game selection method combining ADTree modeling with integer linear programming [32,33]. The only response considered in this approach is to fix the vulnerabilities, and the many aspects (risk, cost, budget constraints, etc.) that define the optimal response plan are not considered. Speicher et al. proposed a Stackelberg programming algorithm that models the game choice problem as a two-person game in which the defender applies mitigation strategies (to minimize the probability of a successful attack) and the attacker tries to counter and maximize his chances of successfully executing the attack [34]. However, in order to improve the efficiency, this method only considers the critical attack path. Zenitani et al. proposed a multi-objective cost-benefit optimization algorithm, which can replace the use of multi-objective optimization algorithms and obtain the optimal solution through a series of iterations [35]. However, this method has only been tested in some networks, and its performance in practice cannot be guaranteed.

The above-mentioned research focuses mainly on balancing benefits and costs, and on constructing the objective function, and the research on the algorithm for selecting the optimal protection strategy has not been discussed in-depth. In addition, there are some other problems, such as the use of indicators that are not consistent with globally accepted standards, and so on. Based on the above research, this paper proposes an optimal security protection strategy selection model based on QLPSO. The model mainly answers the following questions: (1) how to evaluate the security risk of the ICS; (2) how to determine the objective function according to the security risk of the ICS and protection cost; (3) how to choose the optimal protection strategy.

## 3. Model Framework

**Figure 1:**
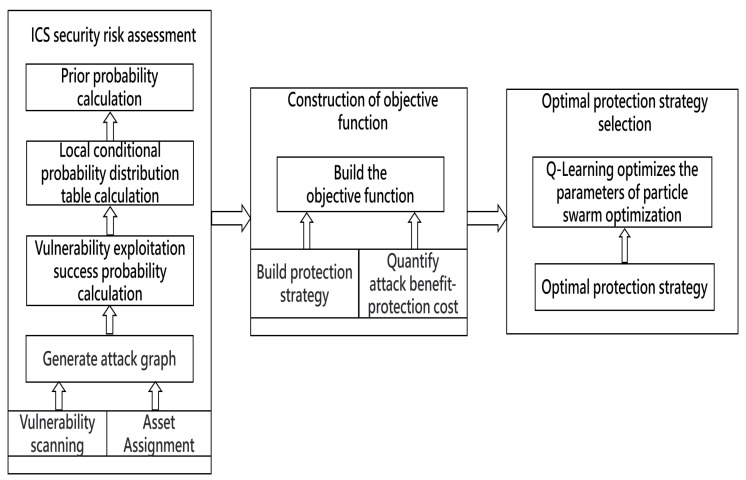
Optimal security protection selection model based on QLPSO.

Our model can be divided into three modules: ICS security risk assessment, construction of objective function, and optimal protection strategy selection. The framework of the model is shown in Figure 1.

(1) ICS security risk assessment: First, we build a Bayesian attack graph based on the network configuration and asset information of the ICS [36], then calculate the exploit success rate of each edge of the attack graph, and construct the local conditional probability distribution (LCPD) table according to the exploit success rate. Finally, the prior probabilities of all attribute nodes being attacked are calculated [37];
(2) Construction of the objective function: Firstly, we construct all possible protection strategies based on the network configuration and asset information of the ICS, then quantify the attack benefit and protection cost, and finally construct the objective function based on both the attack benefit and protection cost;
(3) Optimal protection strategy selection: First, we design the Q-Learning particle swarm optimization algorithm (QLPSO), then solve the objective function using the QLPSO, and finally find the optimal protection strategy.

## 4. ICS Security Risk Assessment

### 4.1. Definition of Bayesian Attack Graph

The Bayesian attack graph is a directed acyclic graph, defined as: BAG=(S,E,A,P), where

(1) $S=\{S_{1},S_{2},\ldots ,S_{n}\}$ is the set of all attribute nodes of the attack graph.
(2) $E=\left\{\ldots ,E_{ij},\ldots \right\}$ is the set of all directed edges of the attack graph, where $E_{ij}$ has two end nodes $S_{i}$ and $S_{j}$, and $S_{i}$ is the parent node and $S_{j}$ the child node.
(3) $A=\left\{A_{1},A_{2},\ldots ,A_{n}\right\}$ means atomic attack. $A_{i}=1$ means that the attack has been launched, otherwise $A_{i}=0$.
(4) $P=\{P\left(S_{1}\right),P\left(S_{2}\right),\ldots ,P\left(S_{n}\right)\}$ is the set of the probabilities that the attribute nodes can be attacked. $P(S_{i})$ indicates the success probability of attribute node $S_{i}$ of being attacked.

The Bayesian attack graph is established by combining the network configuration and asset information, and can be directly constructed using the MulVAL tool [38].

### 4.2. Calculation of Success Probability of Vulnerability Exploitation

We adopt the vulnerability scoring system CVSS (common vulnerability scoring system [39]) to calculate the probability of successful exploitation of each vulnerability, which is given by

(1) $$p\left(S_{i}\right)=2\times AV\times AC\times AU$$

where AV, AC, and AU are the CVSS availability indicators. The specific scores of CVSS indicators are shown in Table 1. AV is the attack route value. The higher the AV, the farther the attacker can launch an attack. AC is the attack complexity value. The higher the AC score, the lower the attack complexity of the attacker. AU is the authentication value. The higher the AU score, the less authentication times the attacker launches attacks. $v_{i}$ represents the exploit between the current node and its parent node.

### 4.3. Calculation of Local Conditional Probability Distribution (LCPD)

**Figure 2:**
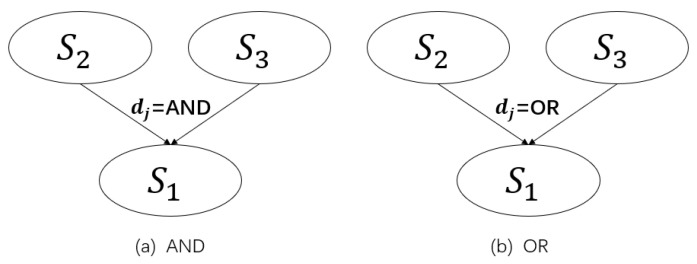
Attribute node and its parent node dependencies.

Conditional probability [40] represents the successful exploitation probability of an attribute node under the influence of its parent node set, denoted by $p(S_{i}|Par\left(S_{i}\right))$. $Par(S_{i})$ refers to the set of parent nodes of the $S_{i}$. The relationship between the attribute node and its parent node is shown in Figure 2. $d_{j}$ represents the respective type of each attribute node, which is divided into two types: AND and OR.

When $d_{j}=\mathrm{AND}$, attribute node $S_{i}$ is only exploited when all its parent nodes are exploited,

(2) $$p\left(S_{i}|\mathrm{Par}\left(S_{i}\right)\right)=\prod_{S_{j}\in \mathrm{Par}\left(S_{i}\right)}p\left(S_{j}\right)$$

When $d_{j}=\mathrm{OR}$, attribute node $S_{i}$ can be exploited when any of its parent nodes are exploited,

(3) $$p\left(S_{i}|\mathrm{Par}\left(S_{i}\right)\right)=1-\prod_{S_{j}\in \mathrm{Par}\left(S_{i}\right)}\left[1-p\left(S_{j}\right)\right]$$

### 4.4. Prior Probability Calculation

The prior probability of an attribute node $S_{i}$ refers to the probability that the attribute node $S_{i}$ can be reached under static conditions. It is expressed as the joint probability of the attribute node $S_{i}$ and all attribute nodes in the path to reach the attribute node $S_{i}$.

The prior probability of attribute node $S_{i}$ is calculated as follows. When $d_{j}=\mathrm{AND}$, attribute node $S_{i}$ is only exploited when all its parent nodes are exploited,

(4) $$P\left(S_{i}\right)=\prod_{S_{j}\in \mathrm{Par}\left(S_{i}\right)}P\left(S_{j}\right)p\left(S_{i}\right)$$

When $d_{j}=\mathrm{OR}$, attribute node $S_{i}$ can be exploited when any of its parent nodes are exploited,

(5) $$P\left(S_{i}\right)=1-\prod_{S_{j}\in \mathrm{Par}\left(S_{i}\right)}\left[1-P\left(S_{j}\right)p\left(S_{i}\right)\right]$$

When the LCPD tables are calculated, the prior probability of each attribute node is equal to the conditional probability of the attribute node multiplied by the conditional probability of its parent node.

## 5. Construction of Objective Function

### 5.1. Protection Strategy

The *M* is a set of protection strategies, denoted as $M=\{M_{1},M_{2},\ldots ,M_{n}\}$, where $M_{i}$ is a protection strategy that can be operated on the attribute node $S_{i}$ to reduce its risk of being attacked. $M_{i}=1$ represents that the protection strategy is enabled, otherwise $M_{i}=0$. When the protection strategy is enabled, the exploit success probability of attribute nodes will be affected, followed by a certain reduction in the exploitation probability. That is

(6) $$p\left(S_{i}|M_{i}=1\right)<p\left(S_{i}|M_{i}=0\right).$$

As a consequence, we have

(7) $$P\left(S_{i}|M\neq 0\right)\leq P\left(S_{i}|M=0\right).$$

Implementing protection strategies will inevitably incur protection costs. The protection cost is represented by $COST=\left\{COST_{1},COST_{2},\ldots ,COST_{n}\right\}$, where $COST_{i}$ represents the cost of implementing the protection strategy $M_{i}$. $COST_{i}$ is defined as [41]:

(8) $$COST_{i}=\omega_{i}\times value\times 100$$

where $\omega_{i}$ is the normalized weight of the protection strategies, and value represents the value of the asset. Therefore, the total protection cost of implementing the protection strategies $M=\{M_{1},M_{2},\ldots ,M_{n}\}$ is given by.

(9) $$C\left(M\right)=\sum_{i=1}^{n}M_{i}COST_{i}.$$

### 5.2. Attack Benefit

The attack benefit of attribute node $S_{i}$ is expressed as $AG(S_{i}$), which refers to the attack benefit obtained by successfully attacking attribute node $S_{i}$. $AG(S_{i}$) can be calculated as

(10) $$AG\left(S_{i}\right)=P\left(S_{i}\right)\times value.$$

In addition, the attack benefit of attribute node $S_{i}$ under the protection strategies *M* is given by

(11) $$AG\left(S_{i}|M\right)=P\left(S_{i}|M\right)\times value.$$

Therefore, the total attack benefit under the protection strategies *M* can be obtained by summing the benefits of all attribute nodes, that is

(12) $$AG\left(M\right)=\sum_{S_{i}\in S}AG\left(S_{i}|M\right).$$

### 5.3. Attack Benefit-Protection Cost Objective Function

Under the above definitions of attack benefit and protection cost, it is easier to illustrate our objective as being to minimize both the attack benefit and the protection cost. Therefore, the objective function can be expressed as

(13) min(δAG(M)+(1−δ)C(M))

subject to

(14) C(M)<B

where δ and 1−δ are the preference weights of attack benefit and defense cost, respectively, and 0≤δ≤1. *B* is the constraint of total protection cost.

## 6. Q-Learning Particle Swarm Optimization Algorithm

### 6.1. Particle Swarm Optimization

In 1995, Kennedy and Eberhardt proposed the PSO algorithm, which is motivated by imitating the foraging activities of birds for solving single-objective optimization problems [42]. The state of each particle *i* in the particle swarm has two fundamental properties: velocity $V_{i}$ and position $X_{i}$, where:

(15) $$V_{i}=\left(V_{i1},V_{i2},\ldots ,V_{iD}\right),i=1,2,\ldots ,N$$

(16) $$X_{i}=\left(X_{i1},X_{i2},\ldots ,\ldots ,X_{iD}\right),i=1,2,\ldots ,N$$

Among them, $V_{ij}$ represents the velocity of the *i*th particle in the *j*th dimension, and $X_{ij}$ represents the position of the *i*th particle in the *j*th dimension. *D* is the dimension.

All particles have memory, which enables them to remember their local optimal position *P*. At the same time, all *P* of different particles will be shared in the whole population, and the optimal *P* is regarded as the global optimal position Gp reached by the population. Based on the obtained *P* and Gp, all particles in the population are updated in position and velocity using the following equations [43].

(17) $$\begin{array}{c} \begin{array}{c} V_{id}^{t+1}=weight\times V_{id}^{t}+Lp\times \mathrm{rand}\left(0,1\right)\times \left(P_{id}^{t}-X_{id}^{t}\right)\\ +Lb\times \mathrm{rand}\left(0,1\right)\times \left(Gp_{d}^{t}-X_{id}^{t}\right) \end{array} \end{array}$$

(18) $$\begin{array}{c} \begin{array}{c} X_{id}^{t+1}=X_{id}^{t}+V_{id}^{t} \end{array} \end{array}$$

Among them, weight is the inertia weight, Lp is the self-learning factor, Lb is the global learning factor, rand (0, 1) is a random number in [0, 1]. $V_{id}^{t}$ represents the *d*th dimensional velocity of the *i*th particle in the *t*th generation. $P_{id}^{t}$ indicates the local optimal particle of the *i*th particle in the *d*th dimension of the *t*th generation. $Gp_{d}^{t}$ represents the globally optimal particle in the *d*th dimension of the *t*th generation. $X_{id}^{t}$ represents the *d*th dimensional position of the *i*th particle in the *t*th generation.

### 6.2. Q-Learning

Q-Learning is a learning method proposed by Watkins in 1989 [44]. In Q-Learning, an agent learns in a ‘trial and error’ way, and the reward guides behavior obtained by interacting with the environment, and the goal is to make the agent obtain the maximum reward.

Q-Learning has four key elements: *Q* table, state, action, and reward. The process of Q-Learning is [45]: the agent selects the action with the largest *Q* value from the *Q* table according to the state. After the action is completed, the state changes, and the reward is determined according to the quality of the state change, and then according to the current state, the next state, actions, and reward values update the *Q* table, in turn.

The updated method of the *Q* table is as follows [46].

(19) $$\begin{array}{c} Q\left(st_{t+1},at_{t+1}\right)=\left(1-\alpha \right)Q\left(st_{t},at_{t}\right)+\alpha \left[R\left(st_{t},at_{t}\right)+\gamma \max_{at}Q\left(st_{t+1},at\right)\right] \end{array}$$

where, $st_{t}$ is the current state, $st_{t+1}$ is the next state. $at_{t}$ is the action taken for the current state, and $at_{t+1}$ is the action taken for the next state. α and γ are the learning rate and discount factor, respectively, R($st_{t}$,$at_{t}$) is the reward generated by the action $at_{t}$ taken by the state $st_{t}$. $max_{at}Q\left(st_{t+1},at\right)$ refers to the maximum *Q* value in the case of state $st_{t+1}$.

**Figure 3:**
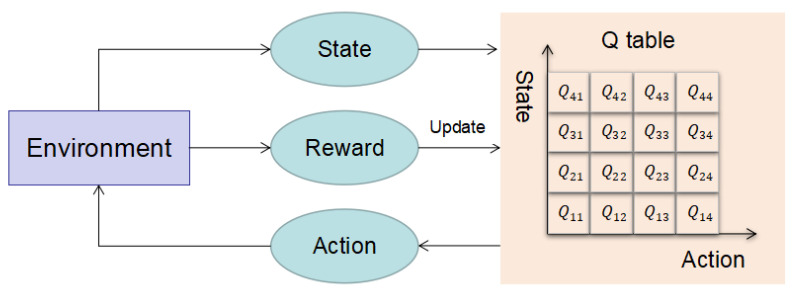
Q-Learning model.

The model of Q-Learning is shown in Figure 3.

### 6.3. Q-Learning Particle Swarm Optimization (QLPSO)

The PSO algorithm can be directly used to solve the optimization objective function Equation (13). However, PSO often falls into local optimum due to its fixed parameters. In this paper, we consider the QLPSO algorithm, which could update the parameters of PSO algorithm through the Q-Learning to avoid the local optimum problem [47].

**Figure 4:**
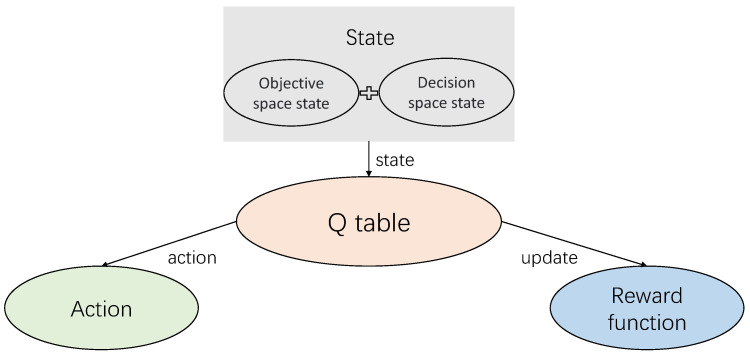
Important elements of the QLPSO algorithm.

The state, action, *Q* table, and reward are also the core elements of the QLPSO algorithm, which are shown in Figure 4.

(1) States

Unlike PSO, which has only one state, QLPSO has two states: objective space state and decision space state. The objective space state needs to consider the relationship between the particle and the global optimal particle position. The decision space needs to consider the relationship between the fitness of the particle and the fitness of the global optimal particle.

The decision space state has four sub-states: DFarthest, DFarther, DNearer, and DNearest. They respectively represent the relative state of the Euclidean distance between the particle and the global optimal position Gp compared with the size of the search space. The objective space state also has four sub-states: the largest fitness difference, the larger fitness difference, the smaller fitness difference, and the smallest fitness difference. They represent the relative state of the particle’s fitness compared to the global optimal fitness and the global worst fitness difference, respectively. In this article, we only need to consider the fitness value between the two solutions.

The specific information of decision space state and objective space state is shown in Table 2 and Table 3 [48].

In Table 2, Rd is the Euclidean distance between a certain particle and the global optimal particle Gp. △R is the range of decision space search. In Table 3, Rf is the fitness, which refers to the fitness difference between a certain particle and the global optimal particle Gp. △F is the difference between the fitness of the global best particle and the fitness of the global worst particle.

(2) Action

There are also four types of actions, which correspond to different parameters of the particle swarm: weight, Lp, and Lb. Different values of weight, Lp and Lb will affect the exploration form of the particle. The larger the weight is, the stronger the global exploration ability and the weaker the local exploration ability. On the contrary, the smaller the weight is, the weaker the global exploration ability and the stronger the local exploration ability. The larger the Lp, the stronger the global exploration ability. The larger the Lb, the stronger the particle convergence ability [49]. The detailed parameter settings are shown in Table 4.

(3) *Q* table

**Figure 5:**
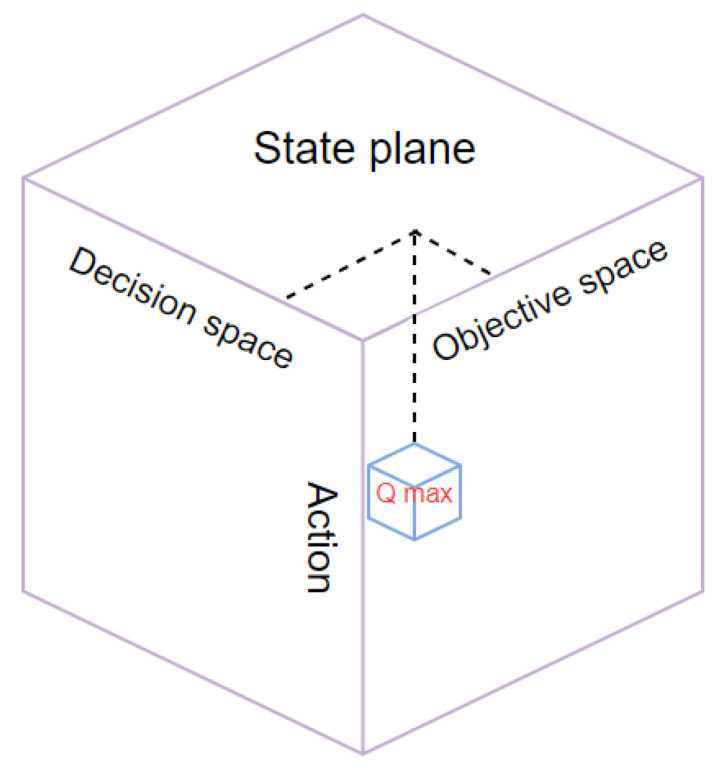
Three-dimensional *Q* table.

Since there are four types of objective space states, decision space states, and actions, the *Q* table of QLPSO is different from the two-dimensional *Q* table used in general Q-Learning. The three-dimensional *Q* table used here is a 4×4×4 three-dimensional *Q* table. The three-dimensional *Q* table is shown in Figure 5. As shown in the figure, we first determine the state of the objective space and the state of the decision space, such as (the closest distance, the smallest fitness difference). Then, according to the state of the objective space and the state of the decision space, the action with the largest *Q* value corresponding to the state is selected.

(4) Rewards

After selecting a certain action, if the fitness value becomes worse, it should be punished. Otherwise, if the fitness value becomes better, it should be rewarded. The reward function defined in this paper is as follows.

(20) $$\begin{array}{c} R=Rf(state+1)-Rf(state) \end{array}$$

where Rf(state) and Rf(state+1) represent the fitness values of the current state and the next state, respectively.

**Figure 6:**
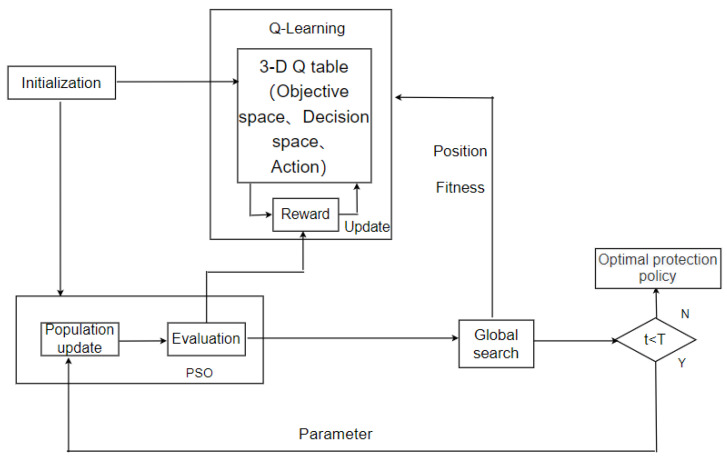
Flowchart of the QLPSO algorithm.

The flowchart of the QLPSO algorithm is shown in Figure 6 and described as follows.

(1) Initialize the population and *Q* table;
(2) The state of each particle is determined according to its position in the objective space and decision space;
(3) The action (parameter) of the particle is determined using the *Q* table;
(4) Update the particles according to the parameters determined in the previous step;
(5) Update the *Q* table according to the reward function;
(6) These steps are repeated for all particles in each generation until the number of iterations is reached.

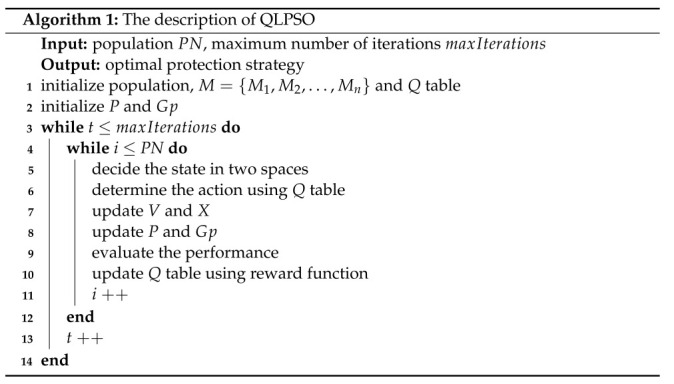


## 7. Experimental

### 7.1. Experimental Scenario

**Figure 7:**
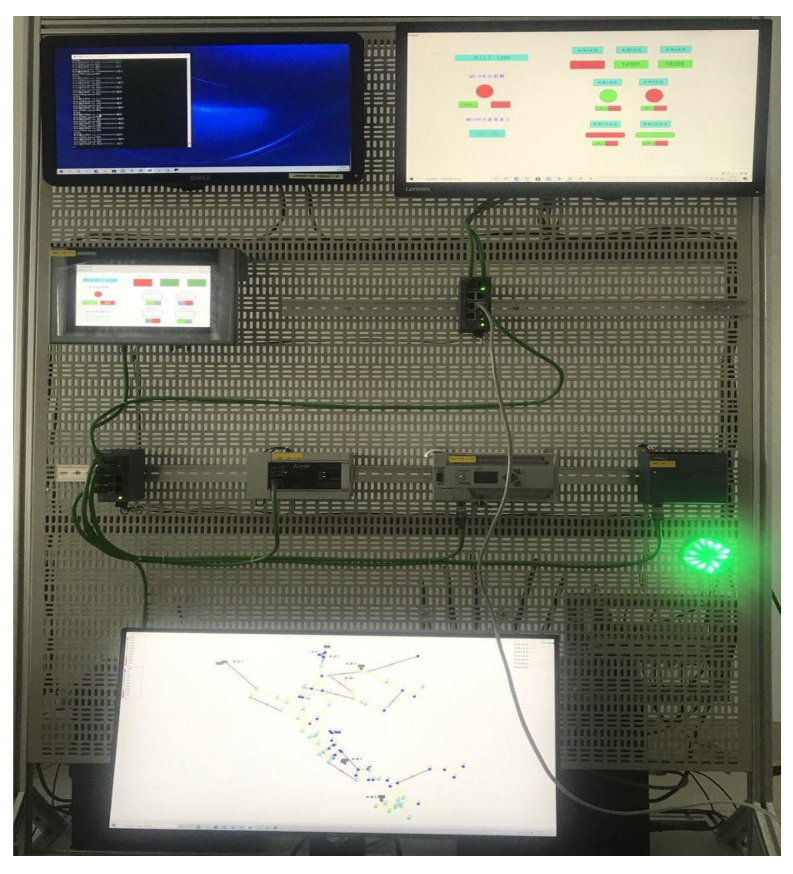
Experimental scene.

The experimental scenario in this paper is a water distribution system, as shown in Figure 7. There are three PLCs in the water distribution system, which respectively control the water pump, the pipeline, and the water tanker in the virtual system. The platform can be used for industrial control security attack and defense drills, security risk assessment, and other related experiments [50].

### 7.2. Generating a Bayesian Attack Graph

**Figure 8:**
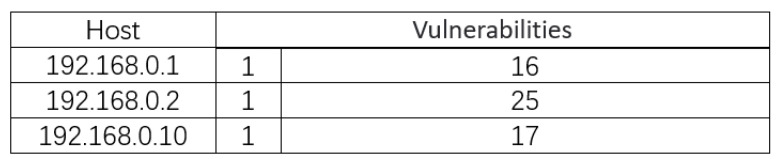
Asset and vulnerability information.

**Figure 9:**
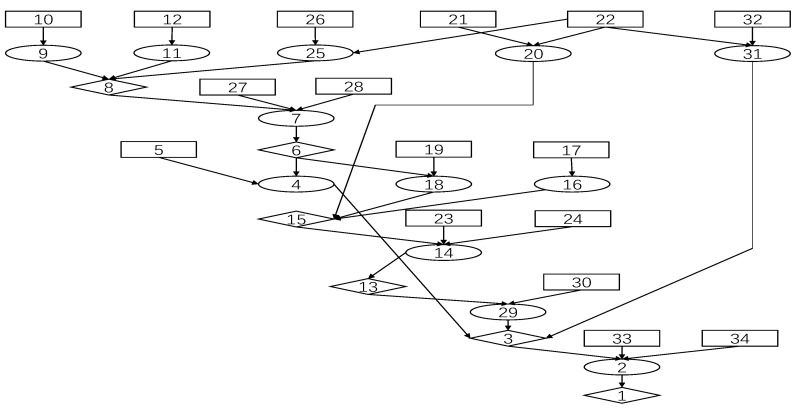
Bayesian attack graph.

Asset and vulnerability information is obtained by scanning assets as shown in Figure 8. The asset consists of three PLCS, each of which has the same vulnerability CVE-1999-0517. At the same time, there is some additional information for each PLC. The attack graph of the water distribution system is generated by using the MulVal. It is shown in Figure 9 and Table 5.

### 7.3. Attack Benefits and Protection Costs

The prior probabilities of all attribute nodes are calculated through the LCPD table, as shown in Table 6. The quantification standard of cost index of protection operation is shown in Table 7 [34]. In Table 7, Pi refers to the impact of the implementation strategy on vulnerability utilization. The specific operation, cost and other details of the protection strategies are shown in Table 8.

### 7.4. Experimental Results

In this paper, assuming the benefits of attack are as important as the costs of protection. The fitness function parameter δ is set to 0.5, and there is no upper limit to the set-up cost *B*.

**Figure 10:**
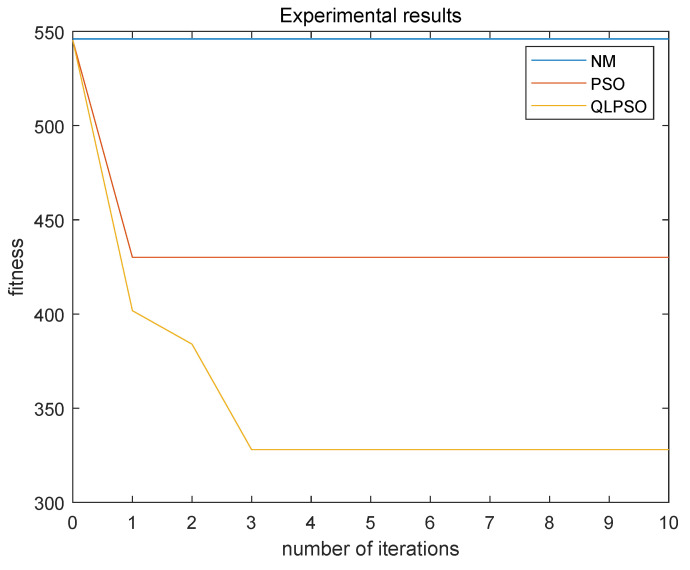
Experimental results.

The experimental results are shown in Figure 10. The blue curve is the experimental result without protection strategies. The fitness value is always at 546.0160, and the attack benefit is very large, which means that without protection strategies, the attacker can very easily launch attacks to obtain attack benefit. The red curve is the experimental result of the ordinary particle swarm. It can be seen that the fitness value is always 430.0326, and the attack benefit plus protection cost is still quite large, indicating that the optimal protection strategy has not been obtained. The yellow curve is the experimental result of the Q-Learning particle swarm. It can be seen that the fitness value tends to be stable at 327.9708, and the attack benefit plus protection cost reaches the minimum value. The experiments show that the Q-Learning particle swarm algorithm found the optimal protection strategy [0 0 0 1 0 0 0 0 0 0 0 0 1 1 0 0 0 1 0 0 0 0 0 0 0 0 0 0 0 0 0 0 1 0 0 0], and the attack benefit plus protection cost reached a minimum of 332.4039.

It is proved that the optimal protection strategy obtained by QLPSO algorithm has a good protection effect and minimizes the attack benefit and protection cost. Therefore, the water distribution system is well protected by the strategy selected through QLPSO.

## 8. Conclusions

Constructing the optimal security protection strategy is of great significance to the ICS. Since the traditional PSO algorithm tends to fall into local optimum when choosing the optimal protection strategy, in this paper, we propose an optimal security protection strategy selection model and Q-Learning particle swarm optimization algorithm. The algorithm can easily select the optimal protection strategy and the experimental results verify the feasibility and effectiveness of our model and the QLPSO algorithm.

## Figures and Tables

**Table 1:** CVSS score.

Index	Rank	Score
	local access	0.395
Access Vector (AV)	adjacent network access	0.646
	network accessible	1.0
	high	0.35
Access complexity (AC)	medium	0.61
	low	0.71
	multiple instances of authentication	0.45
Authentication (AU)	single instance of authentication	0.56
	no authentication	0.704

**Table 2:** Decision space state.

Relative Distance (Rd)	Decision Space State
0≤Rd≤0.25△R	DNearest
0.25△R≤Rd≤0.5△R	DNearer
0.5△R≤Rd≤0.75△R	DFarther
0.75△R≤Rd≤△R	DFarthest

**Table 3:** Objective space state.

Single-Objective Problem Relative Fitness (Rf )	Objective Space State
0≤Rf≤0.25△F	FSmallest
0.25△F≤Rf≤0.5△F	FSmaller
0.5△F≤Rf≤0.75△F	FLarger
0.75△F≤Rf≤△F	FLargest

**Table 4:** Detailed parameter settings of the actions.

Search Status	Weight	*Lp*	*Lb*
Large scale search	1	0.5	0.5
Small scale search	0.8	2	1
Slow search	0.6	1	2
Fast search	0.4	0.5	0.5

**Table 5:** Prior probabilities of each node of the Bayesian attack graph.

Attribute Nodes	Prior Probability	Attribute Nodes	Prior Probability	Attribute Nodes	Priori Probability
$S_{1}$	0.5000	$S_{2}$	0.1812	$S_{3}$	0.5000
$S_{4}$	0.5000	$S_{5}$	1.0000	$S_{6}$	1.0000
$S_{7}$	0.3967	$S_{8}$	0.5000	$S_{9}$	1.0000
$S_{10}$	1.0000	$S_{11}$	1.0000	$S_{12}$	1.0000
$S_{13}$	0.8372	$S_{14}$	0.3257	$S_{15}$	0.5000
$S_{16}$	1.0000	$S_{17}$	1.0000	$S_{18}$	0.5000
$S_{19}$	1.0000	$S_{20}$	0.6793	$S_{21}$	1.0000
$S_{22}$	1.0000	$S_{23}$	1.0000	$S_{24}$	0.9863
$S_{25}$	0.8000	$S_{26}$	1.0000	$S_{27}$	1.0000
$S_{28}$	0.9960	$S_{29}$	0.5814	$S_{30}$	1.0000
$S_{31}$	0.5641	$S_{32}$	1.0000	$S_{33}$	1.0000
$S_{34}$	0.9530				

**Table 6:** Details of the attack graph.

Node	Node Information
1	execCode(192.168.0.1,someUser):0.3623
2	RULE 2 (remote exploit of a server program):0.3623
3	netAccess(192.168.0.1,udp,161):0.906
4	RULE 5 (multi-hop access):0.3173
5	hacl(192.168.0.10,192.168.0.1,udp,161):1.0
6	execCode(192.168.0.10,someUser):0.3967
7	RULE 2 (remote exploit of a server program):0.3967
8	netAccess(192.168.0.10,udp,161):0.992
9	RULE 5 (multi-hop access):0.8
10	hacl(192.168.0.1,192.168.0.10,udp,161):1.0
11	RULE 5 (multi-hop access):0.8
12	hacl(192.168.0.2,192.168.0.10,udp,161):1.0
13	execCode(192.168.0.2,someUser):0.389
14	RULE 2 (remote exploit of a server program):0.389
15	netAccess(192.168.0.2,udp,161):0.9727
16	RULE 5 (multi-hop access):0.8
17	hacl(192.168.0.1,192.168.0.2,udp,161):1.0
18	RULE 5 (multi-hop access):0.3173
19	hacl(192.168.0.10,192.168.0.2,udp,161):1.0
20	RULE 6 (direct network access):0.8
21	hacl(internet,192.168.0.2,udp,161):1.0
22	attackerLocated(internet):1.0
23	networkServiceInfo(192.168.0.2,sun sunos,udp,161,someUser):1.0
24	vulExists(192.168.0.2,CVE-1999-0517,sun sunos,remoteExploit,privEscalation):0.4998
25	RULE 6 (direct network access):0.8
26	hacl(internet,192.168.0.10,udp,161):1.0
27	networkServiceInfo(192.168.0.10, sun sunos,udp,161,someUser):1.0
28	vulExists(192.168.0.10,CVE-1999-0517,sun sunos,remoteExploit,privEscalation):0.4998
29	RULE 5 (multi-hop access):0.3112
30	hacl(192.168.0.2,192.168.0.1,udp,161):1.0
31	RULE 6 (direct network access):0.8
32	hacl(internet,192.168.0.1,udp,161):1.0
33	networkServiceInfo(192.168.0.1,sun sunos,udp,161,someUser):1.0
34	vulExists(192.168.0.1,CVE-1999-0517,sun sunos,remoteExploit,privEscalation):0.4998

**Table 7:** Protection cost quantification standard.

Index	Weight ($\omega_{i}$)
Disable cost	0.357
Disconnect cost	0.286
Patch cost	0.214
Install cost	0.143

**Table 8:** Detailed parameters of the protective strategies.

Protective Strategies	Protective Action	COST	*Pi*
$M_{1}$	Disconnect 192.168.0.1–192.168.0.10	14	0.25
$M_{2}$	Disconnect Disconnect 192.168.0.2–192.168.0.10	14	0.25
$M_{3}$	Disconnect Internet 192.168.0.10	15	0.30
$M_{4}$	Disconnect Internet 192.168.0.2	15	0.30
$M_{5}$	Disconnect Internet	22	0.05
$M_{6}$	Disable Internet	22	0.05
$M_{7}$	Disable Internet direct network access	22	0.05
$M_{8}$	Disconnect Internet 192.168.0.1	15	0.30
$M_{9}$	Disable multi-hop access 192.168.0.10	12	0.10
$M_{10}$	Disable udp	12	0.10
$M_{11}$	Disable direct network access 192.168.0.10	12	0.10
$M_{12}$	Disable direct network access 192.168.0.2	12	0.10
$M_{13}$	Disable direct network access 192.168.0.1	12	0.10
$M_{14}$	Disable netAccess 192.168.0.10	12	0.10
$M_{15}$	Disable networkService 192.168.0.10	18	0.45
$M_{16}$	Patch CVE-1999-0517 192.168.0.10	18	0.45
$M_{17}$	Disable service programs	25	0.20
$M_{18}$	Disconnect 192.168.0.10–192.168.0.1	14	0.25
$M_{19}$	Disable execCode 192.168.0.10	20	0.30
$M_{20}$	Disconnect 192.168.0.10	20	0.30
$M_{21}$	Disconnect 192.168.0.10–192.168.0.2	14	0.25
$M_{22}$	Disconnect 192.168.0.1–192.168.0.2	14	0.25
$M_{23}$	Disable multi-hop access 192.168.0.1	20	0.30
$M_{24}$	Disable multi-hop access 192.168.0.2	20	0.30
$M_{25}$	Disable netAccess 192.168.0.2	20	0.30
$M_{26}$	Disable netAccess	12	0.10
$M_{27}$	Disable networkService 192.168.0.2	14	0.25
$M_{28}$	Patch CVE-1999-0517 192.168.0.2	18	0.45
$M_{29}$	Disable execCode 192.168.0.2	25	0.20
$M_{30}$	Disable multi-hop access	20	0.30
$M_{31}$	Disconnect 192.168.0.2–192.168.0.1	14	0.25
$M_{32}$	Disable netAccess 192.168.0.1	12	0.10
$M_{33}$	Disable service program 192.168.0.1	12	0.10
$M_{34}$	Disable networkService 192.168.0.1	18	0.45
$M_{35}$	Patch CVE-1999-0517 192.168.0.1	18	0.45
$M_{36}$	Install ids	30	0.20

## Data Availability

Not applicable.

## References

[1] Brändle M., Naedele M. (2008). Security for process control systems: An overview. IEEE Secur. Priv..

[2] Fan X., Fan K., Wang Y., Zhou R. Overview of cyber-security of industrial control system. Proceedings of the 2015 international conference on cyber security of smart cities, industrial control system and communications (SSIC).

[3] Wilhoit K. (2013). Who’s really attacking your ICS equipment?. Trend Micro.

[4] Clarke G., Reynders D., Wright E. (2004). Practical Modern SCADA Protocols: DNP3, 60870.5 and Related Systems.

[5] van Schuppen J.H., Boutin O., Kempker P.L., Komenda J., Masopust T., Pambakian N., Ran A.C. (2011). Control of distributed systems: Tutorial and overview. Eur. J. Control.

[6] Babu B., Ijyas T., Muneer P., Varghese J. Security issues in SCADA based industrial control systems. Proceedings of the 2017 2nd International Conference on Anti-Cyber Crimes (ICACC).

[7] Wang Y. SCM/ERP/MES/PCS integration for process enterprise. Proceedings of the 29th Chinese Control Conference.

[8] Li Z., Shahidehpour M., Aminifar F. (2017). Cybersecurity in distributed power systems. Proc. IEEE.

[9] Cruz T., Rosa L., Proença J., Maglaras L., Aubigny M., Lev L., Jiang J., Simões P. (2016). A cybersecurity detection framework for supervisory control and data acquisition systems. IEEE Trans. Ind. Inform..

[10] Chen T.M., Abu-Nimeh S. (2011). Lessons from stuxnet. Computer.

[11] Sun C.C., Hahn A., Liu C.C. (2018). Cyber security of a power grid: State-of-the-art. Int. J. Electr. Power Energy Syst..

[12] Zhao D., Wang L., Wang Z., Xiao G. (2019). Virus propagation and patch distribution in multiplex networks: Modeling, analysis, and optimal allocation. IEEE Trans. Inf. Forensics Secur..

[13] Stouffer K., Falco J., Scarfone K. (2011). Guide to industrial control systems (ICS) security. NIST Spec. Publ..

[14] David A. (2007). Multiple Efforts to Secure Control Systems Are under Way, But Challenges Remain.

[15] Zhao D., Xiao G., Wang Z., Wang L., Xu L. (2021). Minimum dominating set of multiplex networks: Definition, application, and identification. IEEE Trans. Syst. Man Cybern. Syst..

[16] Shameli-Sendi A., Desfossez J., Dagenais M., Jabbarifar M. (2013). A Retroactive-Burst Framework for Automated Intrusion Response System. J. Comput. Netw. Commun..

[17] Deb K., Pratap A., Agarwal S., Meyarivan T. (2002). A fast and elitist multiobjective genetic algorithm: NSGA-II. IEEE Trans. Evol. Comput..

[18] Gonzalez-Granadillo G., Garcia-Alfaro J., Alvarez E., El-Barbori M., Debar H. (2015). Selecting optimal countermeasures for attacks against critical systems using the attack volume model and the RORI index. Comput. Electr. Eng..

[19] Miehling E., Rasouli M., Teneketzis D. Optimal defense policies for partially observable spreading processes on Bayesian attack graphs. Proceedings of the Second ACM Workshop on Moving Target Defense.

[20] Jaquith A. (2007). Security Metrics: Replacing Fear, Uncertainty, and Doubt.

[21] Bandyopadhyay S., Saha S. (2013). Some single-and multiobjective optimization techniques. Unsupervised Classification.

[22] Poolsappasit N., Dewri R., Ray I. (2011). Dynamic security risk management using bayesian attack graphs. IEEE Trans. Dependable Secur. Comput..

[23] Yigit B., Gür G., Alagöz F. Cost-aware network hardening with limited budget using compact attack graphs. Proceedings of the 2014 IEEE Military Communications Conference.

[24] Lei C., Ma D.H., Zhang H.Q. (2017). Optimal strategy selection for moving target defense based on Markov game. IEEE Access.

[25] Herold N., Wachs M., Posselt S.A., Carle G. (2016). An optimal metric-aware response selection strategy for intrusion response systems. Proceedings of the International Symposium on Foundations and Practice of Security.

[26] Butler S.A. Security attribute evaluation method: A cost-benefit approach. Proceedings of the 24th International Conference on Software Engineering.

[27] Butler S.A., Fischbeck P. Multi-attribute risk assessment. Proceedings of the Symposium on Requirements Engineering for Information Security.

[28] Roy A., Kim D.S., Trivedi K.S. Scalable optimal countermeasure selection using implicit enumeration on attack countermeasure trees. Proceedings of the IEEE/IFIP International Conference on Dependable Systems and Networks (DSN 2012).

[29] Viduto V., Maple C., Huang W., López-Peréz D. (2012). A novel risk assessment and optimisation model for a multi-objective network security countermeasure selection problem. Decis. Support Syst..

[30] Dewri R., Ray I., Poolsappasit N., Whitley D. (2012). Optimal security hardening on attack tree models of networks: A cost-benefit analysis. Int. J. Inf. Secur..

[31] Wang S., Zhang Z., Kadobayashi Y. (2013). Exploring attack graph for cost-benefit security hardening: A probabilistic approach. Comput. Secur..

[32] Kordy B., Wideł W. (2017). How well can I secure my system?. Proceedings of the International Conference on Integrated Formal Methods.

[33] Fila B., Wideł W. Exploiting attack–defense trees to find an optimal set of countermeasures. Proceedings of the 2020 IEEE 33rd Computer Security Foundations Symposium (CSF).

[34] Speicher P., Steinmetz M., Künnemann R., Simeonovski M., Pellegrino G., Hoffmann J., Backes M. Formally reasoning about the cost and efficacy of securing the email infrastructure. Proceedings of the 2018 IEEE European Symposium on Security and Privacy (EuroS&P).

[35] Zenitani K. (2022). A multi-objective cost–benefit optimization algorithm for network hardening. Int. J. Inf. Secur..

[36] Frigault M., Wang L. Measuring network security using bayesian network-based attack graphs. Proceedings of the 2008 32nd Annual IEEE International Computer Software and Applications Conference.

[37] Liu Y., Man H. Network vulnerability assessment using Bayesian networks. Proceedings of the Data Mining, Intrusion Detection, Information Assurance, and Data Networks Security 2005.

[38] Ou X., Govindavajhala S., Appel A.W. MulVAL: A Logic-based Network Security Analyzer. Proceedings of the USENIX Security Symposium.

[39] Mell P., Scarfone K., Romanosky S. (2006). Common vulnerability scoring system. IEEE Secur. Priv..

[40] Gao N., Gao L., He Y., Lei Y., Gao Q. (2016). Dynamic security risk assessment model based on Bayesian attack graph. J. Sichuan Univ. (Eng. Sci. Ed.).

[41] Gao N., Gao L., Yiyue H.E., Wang F. (2016). Optimal security hardening measures selection model based on Bayesian attack graph. Comput. Eng. Appl..

[42] Kennedy J., Eberhart R. Particle swarm optimization. Proceedings of the ICNN’95-International Conference on Neural Networks.

[43] Clerc M. (2010). Particle Swarm Optimization.

[44] Jang B., Kim M., Harerimana G., Kim J.W. (2019). Q-learning algorithms: A comprehensive classification and applications. IEEE Access.

[45] Watkins C.J., Dayan P. (1992). Q-learning. Mach. Learn..

[46] Clifton J., Laber E. (2020). Q-learning: Theory and applications. Annu. Rev. Stat. Its Appl..

[47] Liu Y., Lu H., Cheng S., Shi Y. An adaptive online parameter control algorithm for particle swarm optimization based on reinforcement learning. Proceedings of the 2019 IEEE Congress on Evolutionary Computation (CEC).

[48] Abed-Alguni B.H., Paul D.J., Chalup S.K., Henskens F.A. (2016). A comparison study of cooperative Q-learning algorithms for independent learners. Int. J. Artif. Intell..

[49] Meerza S.I.A., Islam M., Uzzal M.M. Q-learning based particle swarm optimization algorithm for optimal path planning of swarm of mobile robots. Proceedings of the 2019 1st International Conference on Advances in Science, Engineering and Robotics Technology (ICASERT).

[50] Xu L., Wang B., Wu X., Zhao D., Zhang L., Wang Z. (2021). Detecting Semantic Attack in SCADA System: A Behavioral Model Based on Secondary Labeling of States-Duration Evolution Graph. IEEE Trans. Netw. Sci. Eng..

